# SPINK4 promotes colorectal cancer cell proliferation and inhibits ferroptosis

**DOI:** 10.1186/s12876-023-02734-2

**Published:** 2023-04-03

**Authors:** Bang-li Hu, Yi-xin Yin, Ke-zhi Li, Si-qi Li, Zhao Li

**Affiliations:** Department of Research, Guangxi Medical University Cancer Hospital, No. 71 Hedi Road, Nanning, 530021 Guangxi PR China

**Keywords:** Colorectal cancer, SPINK4, Ferroptosis, Mouse model

## Abstract

**Background:**

Little is known about the role of serine peptidase inhibitor Kazal type 4 (SPINK4) in colorectal cancer (CRC) and ferroptosis. Therefore, this study aimed to determine the effect of SPINK4 on CRC pathogenesis and ferroptosis.

**Methods:**

SPINK4 expression was analyzed in public datasets and examined using immunohistochemistry. The biological function of SPINK4 in CRC cell lines and its effect on ferroptosis were tested. An immunofluorescence assay was performed to determine the location of SPINK4 in cells, and mouse models were established to determine the effects of SPINK4 in vivo.

**Results:**

CRC datasets and clinical samples analysis revealed that SPINK4 mRNA and protein levels were significantly reduced in CRC tissues compared to control tissues (*P *< 0.05). Two CRC cell lines (HCT116 and LoVo) were selected, and the in vitro and in vivo experiments showed that overexpression of SPINK4 greatly promotes the proliferation and metastasis of CRC cells and tumor growth (*P* < 0.05). The immunofluorescence assay indicated that SPINK4 is mainly located in the nucleoplasm and nucleus of CRC cells. Furthermore, SPINK4 expression was reduced after cell ferroptosis induced by Erastin, and overexpression of SPINK4 greatly inhibited ferroptosis in CRC cells. The results of mouse model further demonstrated that SPINK4 overexpression inhibited CRC cell ferroptosis and facilitated tumor growth.

**Conclusions:**

SPINK4 was decreased in CRC tissues and promoted cell proliferation and metastasis; overexpression of SPINK4 inhibited CRC cell ferroptosis.

**Supplementary Information:**

The online version contains supplementary material available at 10.1186/s12876-023-02734-2.

## Introduction

Cancer of the colon (CRC) is one of the most common and deadly cancers in the world, and while mortality rates have been declining in recent years, recent epidemiological data have shown that the incidence of the disease has been on the rise [1, 2]. Chemotherapy is the main therapy used for patients with CRC who undergo surgery or those at an advanced stage, however the efficacy of this treatment is subject to chemoresistance or toxic side effects [3]. Thus, many studies have endeavored to find more efficient therapies and reduce side effects. Ferroptosis, a newly discovered type of regulated cell death, has emerged as a potential treatment strategy for a variety of diseases [4, 5]. Ferroptosis is different from apoptosis or necroptosis in terms of morphology and biochemistry and can be induced via small molecules such as Erastin, sulfasalazine, and sorafenib [4, 6]. There has been evidence that ferroptosis has been associated with a wide variety of diseases, including tumors, and is associated with chemoresistance as well as the ability of immunotherapy to inhibit tumor growth, indicating that it may be a promising treatment strategy for CRC [7–9].

Contrary to other types of programmed cell death, ferroptosis is characterized by a fatal accumulation of lipid reactive oxygen species (ROS) that is iron-dependent [10]. ACSL4, HSPA5, P53RRA, iron metabolism, and lipid metabolism are only a few of the genes and pathways that have been discovered to influence ferroptosis [11–13]. However, the precise regulatory network of ferroptosis is still mostly unclear and needs to be clarified. Human goblet cells have high levels of the peptide serine peptidase inhibitor Kazal type 4 (SPINK4), which is present in the gastrointestinal system. A recent study indicated that SPINK4 expression was reduced in CRC compared to that in normal tissues and found to be associated with survival in CRC patients, supporting our earlier findings that the serum level of SPINK4 is associated with CRC [14].

In a recent study, it was discovered that SPINK4 expression was lower in CRC than in normal tissues and was related to patient survival [15]. Additionally, in rectal cancer patients receiving concomitant chemoradiotherapy, elevated SPINK4 expression predicts a poor treatment response [16]. These findings showed a strong correlation between SPINK4 and the treatment response in CRC patients. However, there is still no clear indication that SPINK4 participates in the regulation of ferroptosis in CRC. In the present study, we explored the role of SPINK4 in Erastin-induced ferroptosis in CRC using in vivo and in vitro experiments. Our results emphasize the possibility of the role of SPINK4 in the pathogenesis of CRC, and suggest that SPINK4 acts as a key coordinative agent of ferroptosis in CRC.

## Materials and methods

### Antibodies and reagents

These antibodies and reagents were used in the present study: Erastin was purchased from Selleck Chemicals (No. E7781, Houston, TX, USA). Primary antibodies against SPINK4 were purchased from LEAD-BIO (No. 3998, Guangzhou, China); Primary antibodies against GPX4 was purchased from Abcam (No.125066, Cambridge, MA, USA, USA); β-actin was purchased from Cell Signaling Technology (No. 3700, Danvers, MA); HRP-labeled secondary antibody conjugates were purchased from Molecular Probes (Thermo, USA); Glutathione (GSH) Assay kits was purchased from Comim (No. GSH-1-W, Suzhou, China), 4ʹ,6-Diamidino-2-phenylindole (DAPI) was purchased from Sigma-Aldrich (No. D9542, St. Louis, MO, USA).

### Tissue samples collection and immunohistochemistry

Approximately twenty colon cancer tissues and their corresponding adjacent normal tissues were collected from the Guangxi Medical University Cancer Hospital (Nanning, China) between January 2016 and December 2017. The tissues were histologically tested and confirmed to be colon cancer tissue with no severe major organ dysfunction, and no prior cancer chemotherapy. An immunohistochemistry (IHC) assay and image analysis for SPINK4 were performed as previously described [17, 18].

### Analysis of expression of SPINK4 in datasets

Eight GEO datasets, including GSE39582 (582 samples), GSE106582 (194 samples), GSE83889 (136 samples), GSE50421 (49 samples), GSE21510 (148 samples), GSE81558 (51 samples), GSE21815 (141 samples) and GSE32323 (44 samples) were downloaded from the GEO database and the expression of SPINK4 in CRC tissues and the adjacent normal tissues was analyzed. The TCGA-COADREAD dataset (colon and rectal cancer) was obtained from TCGA database. The value of SPINK4 in each dataset was normalized using the log2 transformation prior to analysis.

### Cell line and culture

Four human CRC cell lines (HCT116, LoVo, RKO, and SW480) and one human colon mucosal epithelial cell line (NCM460) purchased from the Chinese Academy of Sciences (Shanghai, China) were cultured in Dulbecco's modified Eagle’s medium (DME) (Gibco, Waltham, USA) supplemented with 10% fetal bovine serum (FBS; Invitrogen, Carlsbad, CA, USA) and 100 mg/mL penicillin/streptomycin (Life Technologies). All the cell lines were cultured at 37 °C and 5% CO2.

### Cell proliferation, migration, and apoptosis assay

Cell proliferation was evaluated using the Cell Counting Kit-8 (CCK-8) assay (No. 96992, Sigma) according to the manufacturer’s instructions. Cell metastasis was assessed using a wound healing assay as previously described [17].

### Induction of cells ferroptosis and GSH and GPX4 levels assay

Erastin was dissolved in 5% dimethyl sulfoxide (DMSO) at a concentration of 40 μM and stored in a dark-colored bottle at -20 °C. Ferroptosis was induced in CRC cells using an Erastin treatment for 48 h, and intracellular GSH and GPX4 levels were used to measure ferroptosis. GSH levels were determined using the corresponding assay kit, according to the manufacturer’s instructions. The results were immediately measured using a colorimetric microplate reader (optical density = 593 nm). GPX4 levels were tested using a western blot assay.

### Cell transfection

The pcDNA3.1-SPINK4 (OE-SPINK4) and pcDNA3.1 empty vector (NC) were chemically synthesized by Hunan Fenghui Biotechnology Co., Ltd. (Hunan, China) and transfected into LoVo and HCT116 cells prior to treatment using Lipofectamine 3000 (Invitrogen, Carlsbad, CA, USA) following the manufacturer's instructions. Further experiments were conducted after 24 h of transfection.

### RNA extraction and RT-PCR assay

RNA extraction and RT-PCR assay was performed as pervious described [19]. Primer sequences of SPINK4 are as follows: Forward:5’—GAC ATT TCA GGG AGG GG AC A-3’, reverse:5’—TGG TTC CCT GTC CTG ATC AC-3’; GAPDH forward:5’-GGA CCT CAT GGC CTA CAT GG-3’, reverse:5’-TAG GGC CTC TCT TGC TCA GT-3’. The relative expression of SPINK4 was determined using the qTOWER® 3 Real-Time PCR (qPCR) system (Analytik Jena, Carlsbad, Germany) and calculated using the 2^−ΔΔCT^ method.

### Western blot assay

Western blot assay was performed as our pervious described [20]. Blots were probed with primary antibodies against SPINK4 (1:1000) or GPX4 (1:1000) at 4 °C overnight, incubated with second antibody horseradish peroxidase (HRP)-coupled goat anti-rabbit IgG (1:500) at room temperature for 1 h. The β-actin was employed as the internal reference for the images.

### Immunofluorescence assay

The immunofluorescence assays were conducted on slides with cells fixed with 4% paraformaldehyde for SPINK4 (1:1000). The secondary antibody used was goat anti-rabbit Alexa Fluor 555 (1:1000; Beyotime, China). A double immunofluorescence assay was performed according to the manufacturer’s instructions. DAPI was used to stain the nuclei of the cells. Fluorescence images were obtained using a confocal laser scanning microscope (Zeiss LSM 980, Airyscan 2).

### Animals experiment

Animal experimental were approved by the Ethics Committee of Animal Experiments of Guangxi Medical University. All procedures were performed in accordance with the institutional guidelines. Sixteen nude mice (4-week-old) were randomly allocated into four groups: NC group (cells with empty vector), OE group (cells with OE-SPINK4), ERA group (Erastin injection), and OE + ERA group (cells with OE-SPINK4 plus Erastin injection), with four mice in each group. HCT116 cells (5 × 10^6^ cells per mouse) transfected with or without OE-SPINK4 were injected subcutaneously into the right posterior flanks of mice to establish subcutaneous tumors. Seven days after injection, mice were randomly allocated into groups and treated with Erastin (30 mg/kg intraperitoneal injection, twice every other day) for two weeks. Four weeks after the start of treatment, all mice were euthanized, tumors were removed, and the tumor size was measured using calipers. The tumors were then collected, cut into pieces of similar size and stored at -80 °C for subsequent experiments. Mice were euthanized as previously described [21] and were deeply anesthetized via an intraperitoneal injection of pentobarbital at a dose of 60 mg/kg. The animals were then quickly sacrificed by dislocating the neck.

### Statistical analysis

Statistical analysis was performed using R software (version 3.6.5). Data are expressed as means ± standard deviation (SD). Statistical significance was determined via the two-sided Student's t-test or analysis of variance (ANOVA), and *P* value < 0.05 was considered statistically significant.

## Results

### Expression of SPINK4 was decreased in CRC tissues

**Fig. 1:**
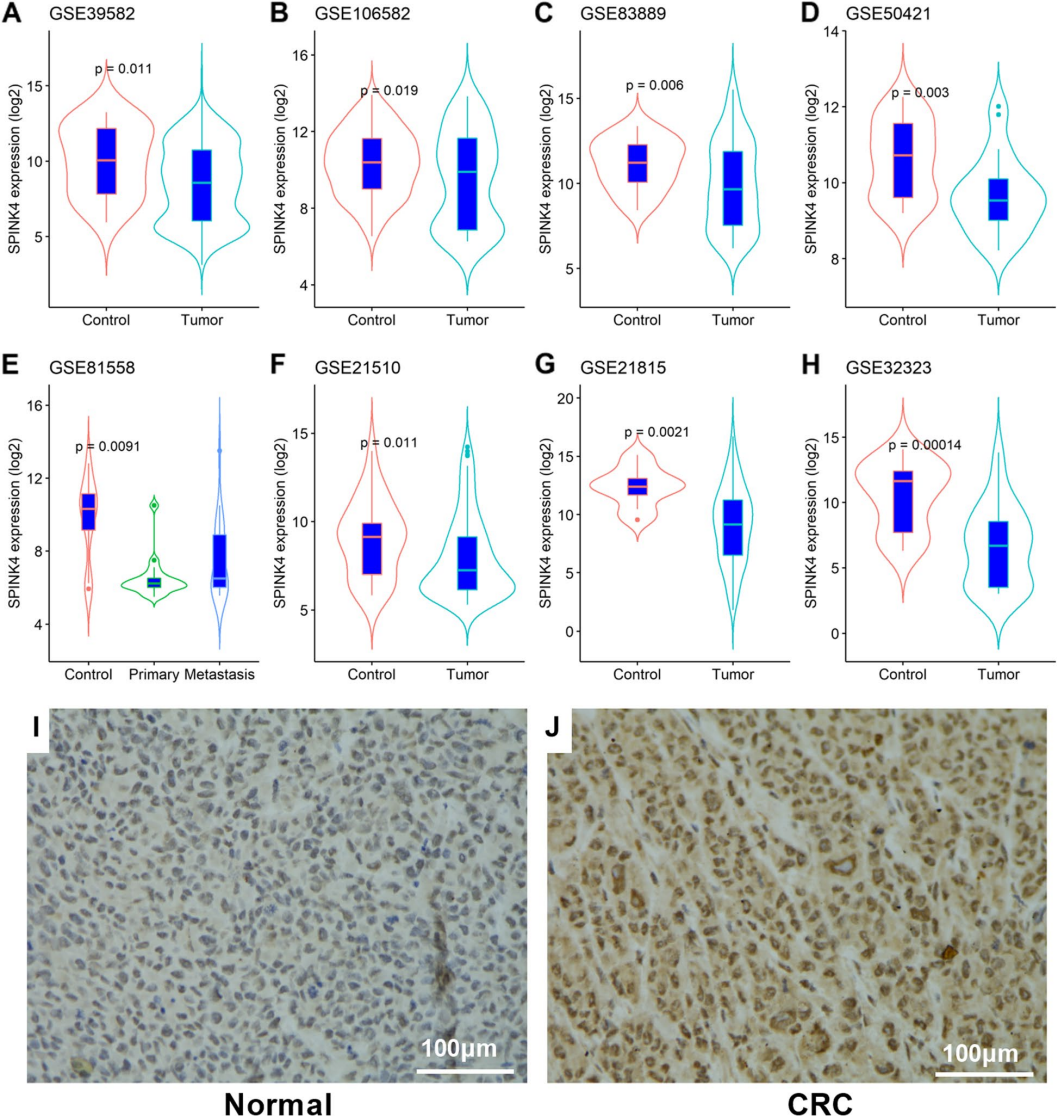
Expression of SPINK4 in CRC and control colon tissues. **A**-**H** Comparison of SPINK4 expression between CRC tissues and control tissues in eight GEO datasets; (**I**-**L**) IHC showed the SPINK4 expression in (**I**) adjacent normal tissues and (**J**) CRC tissues

According to the GTEx database, SPINK4 is specifically overexpressed in normal colon tissue; a previous study [15] and TCGA database indicated that SPINK4 expression was reduced in colon cancer tissues compared with normal tissues. Based on the results of eight GEO datasets, we found that the expression of SPINK4 mRNA was notably decreased in CRC tissues compared to that in control tissues (*P* < 0.05, Fig. 1A-H). Using IHC, we observed that SPINK4 protein levels were reduced in CRC tissues compared with the adjacent normal controls (*P* < 0.05, Fig. 1I-J). These results suggest that SPINK4 expression is reduced in CRC tissues.

### Overexpression of SPINK4 promoted CRC cells proliferation and metastasis

**Fig. 2:**
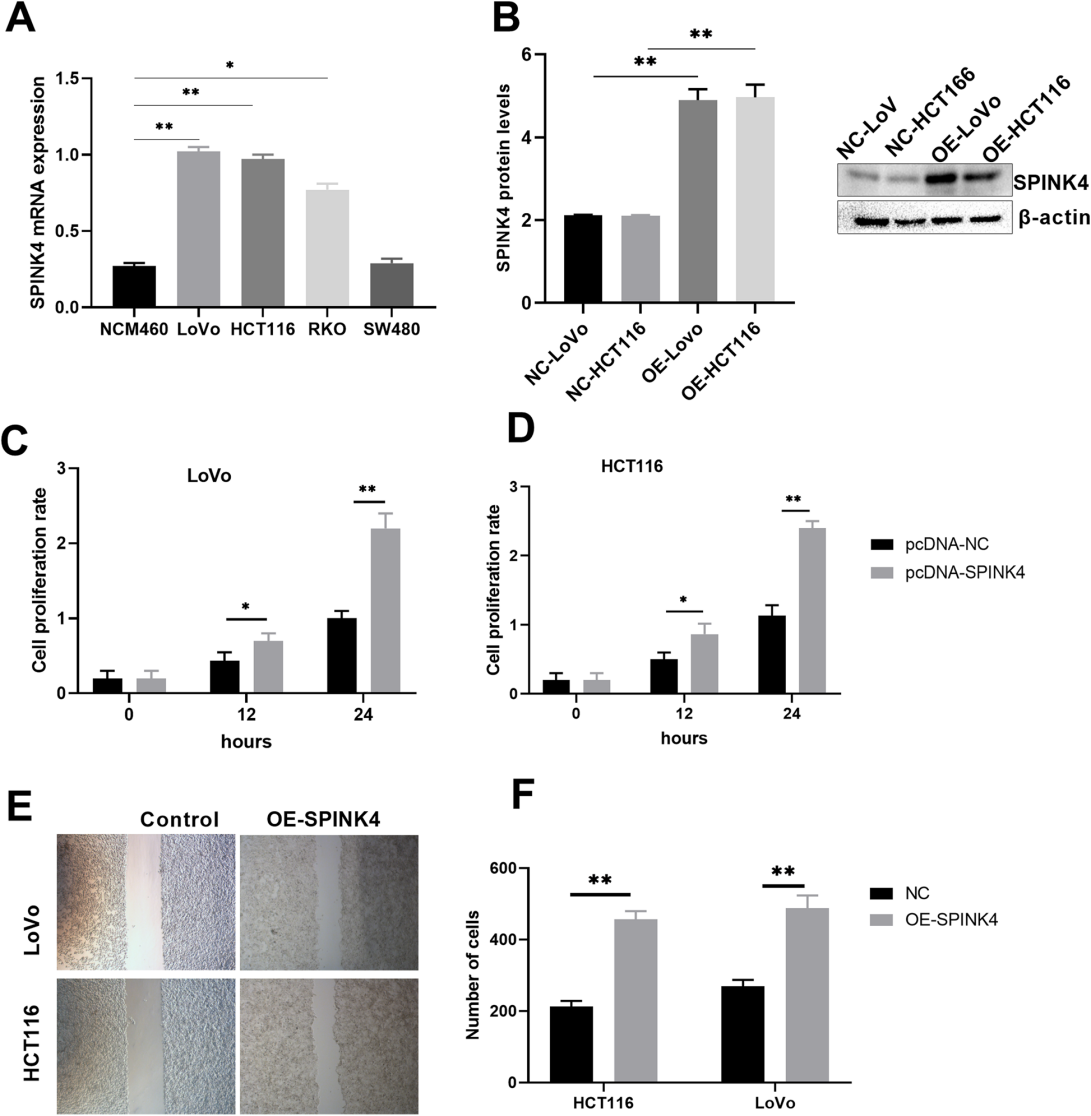
Overexpression of SPINK4 promoted CRC cells proliferation and metastasis. **A** The mRNA expression of SPINK4 in four CRC cells; **B** Overexpression of SPINK4 protein levels in LoVo cells and HCT1116 cells; (**C**, **D**) Overexpression of SPINK4 promoted CRC cells proliferation; (**E**, **F**) Overexpression of SPINK4 promoted CRC cells metastasis. *P < 0.05; **P < 0.01

To further determine the biological function of SPINK4 in CRC, we examined SPINK4 expression in four CRC cell lines (HCT116, LoVo, RKO, and SW480) and a human colon mucosal epithelial cell line (NCM460), and found that SPINK4 mRNA was significantly higher in the LoVo and HCT116 cell lines when compared to the NCM460 cells, however no significant difference was observed between SW480 and NCM460 cells (Fig. 2A). Therefore, we selected LoVo and HCT116 cells for the following experiments. Next, we transfected pcDNA3.1-SPINK4 into LoVo and HCT116 cells to establish cell lines that overexpress SPINK4 (LoVo -pcSPINK4 and HCT116- pcSPINK4) (Fig. 2B).

We then determined the effects of SPINK4 overexpression on the proliferation and metastasis of these two cell lines. The CCK8 assay indicated that overexpression of SPINK4 significantly facilitated the proliferation of LoVo and HCT116 cells (Fig. 2C, D). The wound healing assay indicated that the overexpression of SPINK4 greatly promoted the metastatic ability of the two CRC cell lines (Fig. 2E, F). Overall, these results suggest that SPINK4 overexpression promotes CRC cell proliferation and metastasis.

### SPINK4 was decreased in colon cancer cells with ferroptosis induced by Erastin

**Fig. 3:**
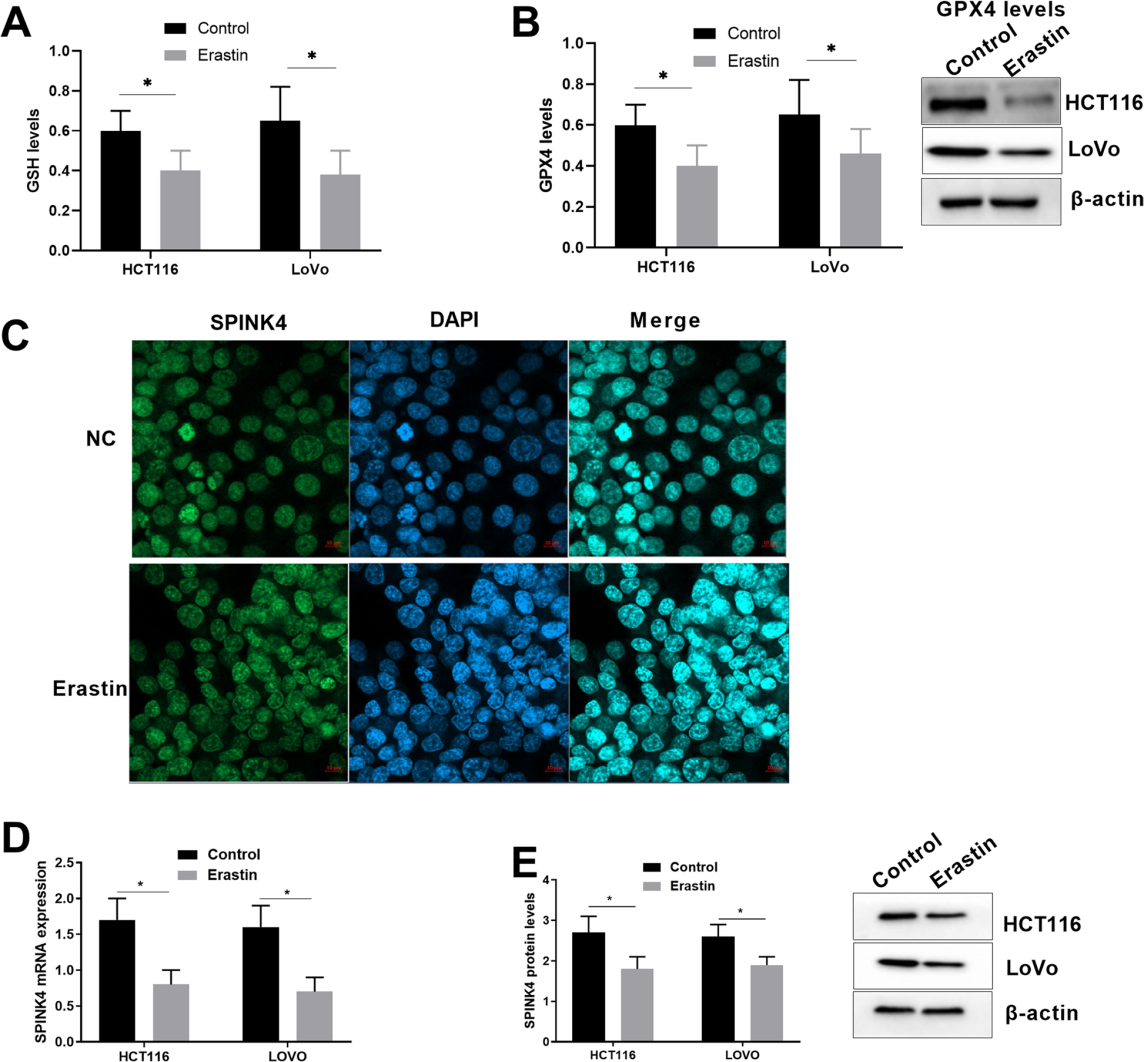
Decreased of SPINK4 after inducing ferroptosis by Erastin. **A**, **B** Ferroptosis developed (GSH and GPX4 levels) in LoVo and HCT166 cells induced by Erastin; **C** SPINK4 protein expression in colon cells by immunofluorescence assay; **D** SPINK4 mRNA expression in CRC cells induced ferroptosis. **E** SPINK4 protein levels in CRC cells induced ferroptosis. *P < 0.05; **P < 0.01

To determine the role of SPINK4 in the ferroptotic process of CRC cells, HCT116 and LoVo cells were treated with Erastin, and the results indicated that ferroptosis was induced in CRC cells based on the decrease in GSH and GPX4 levels (Fig. 3A, B). Using a cell immunofluorescence assay, we found that the SPINK4 protein was mainly located in the nucleoplasm and nucleus, and after treatment with Erastin, the expression of the SPINK4 protein remained in the nucleus of both HCT116 and LoVo cells (Fig. 3C). RT-PCR and western blot assays demonstrated that the SPINK4 mRNA and protein expression levels were reduced after ferroptosis (Fig. 3D, E). These results suggest that SPINK4 is involved in ferroptosis of CRC cells.

### Overexpression of SPINK4 inhibited cell ferroptosis

**Fig. 4:**
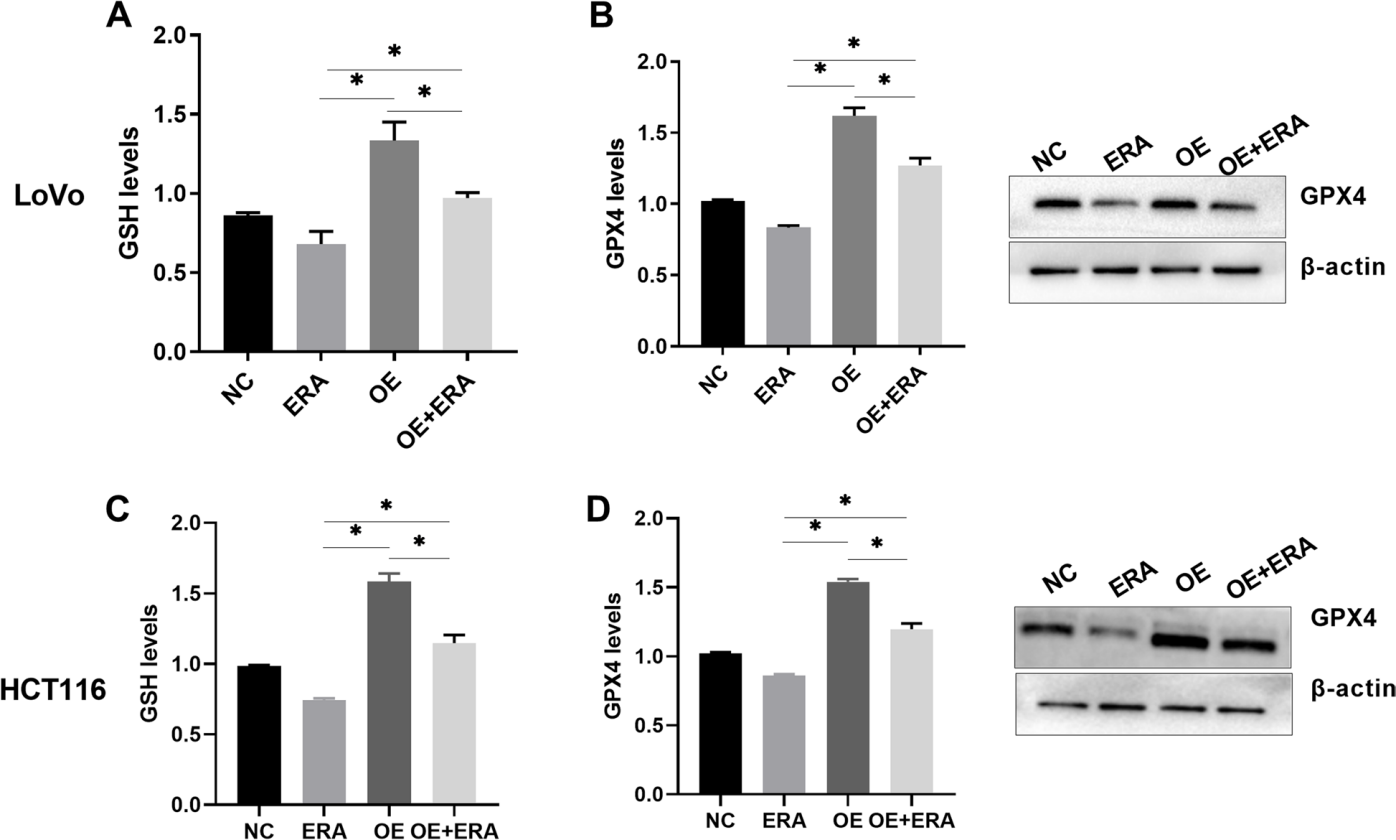
Overexpression of SPINK4 inhibited CRC cells ferroptosis (**A**) Comparisons of GSH levels after LoVo cells treatment with Erastin or overexpression of SPINK4; (**B**) Comparisons of GPX4 levels after LoVo cells treatment with Erastin or overexpression of SPINK4; (**C**) Comparisons of GSH levels after HCT116 cells treatment with Erastin or overexpression of SPINK4; (**D**) Comparisons of GPX4 levels after HCT116 cells treatment with Erastin or overexpression of SPINK4. NC: controls; OE: overexpression of SPINK4; ERA: Erastin; *P < 0.05

To further examine the role of SPINK4 in the ferroptosis of CRC cells, HCT116 and LoVo cells were transfected with pcDNA3.1-SPINK4 in order to facilitate the overexpression of SPINK4, and then treated with Erastin. GSH and GPX4 expression was found to be increased in cells overexpressing SPINK4 compared to that in cells without an overexpression of SPINK4 (Fig. 4A-D). These results prove that SPINK4 overexpression inhibits ferroptosis in CRC cells.

### SPINK4 inhibits ferroptosis in vivo

**Fig. 5:**
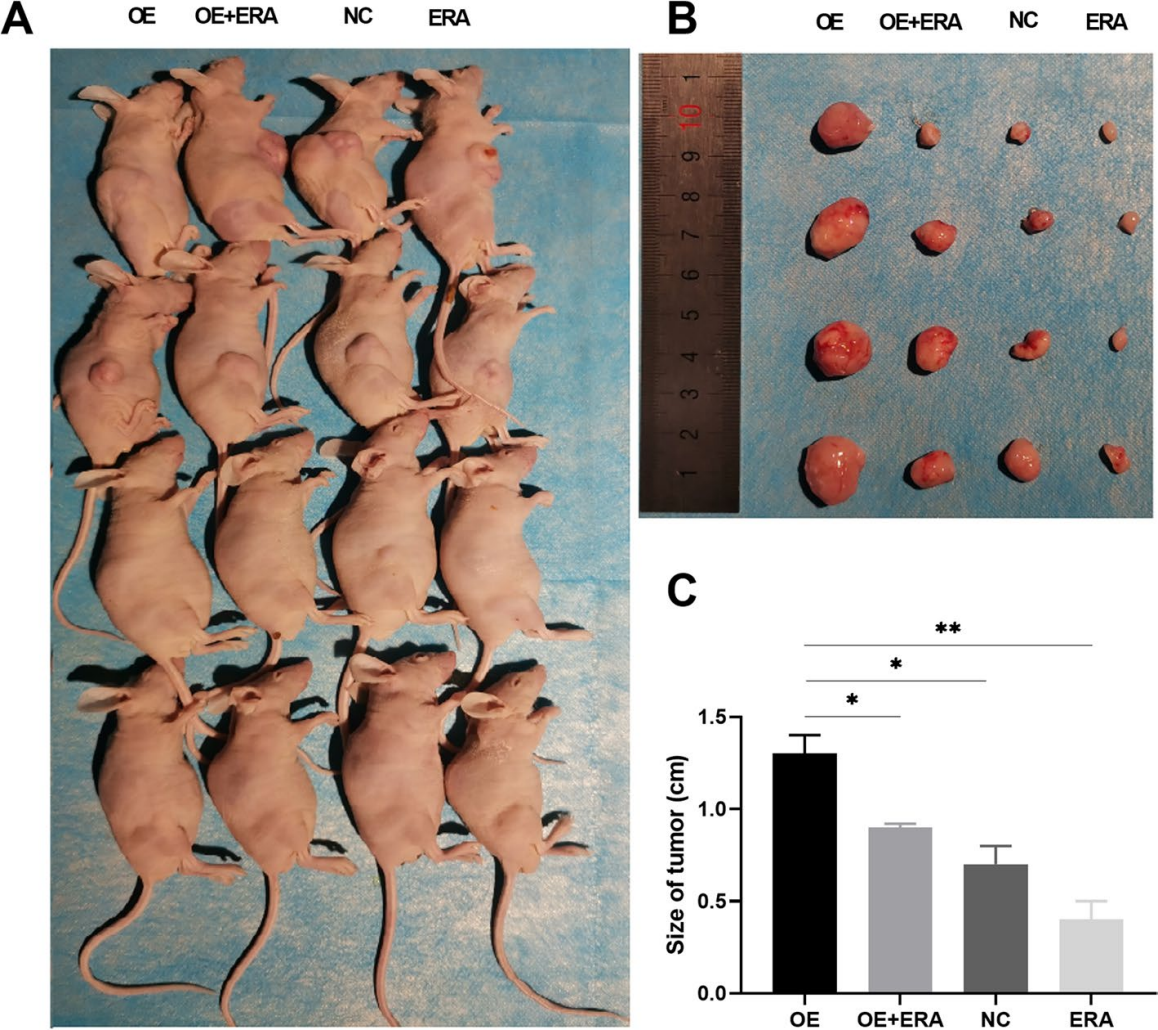
SPINK4 inhibits ferroptosis in vivo. Sixteen mice were randomly divided into four groups with four animals each with comparable mean body weight. Mice of four groups were treated with OE-SPINK4 (OE), OE-SPINK4 + Erastin (OE + ERA), Empty vector controls (NC) and Erastin (ERA). **A**-**C** Size of tumor in four groups. *P < 0.05; **P < 0.01

To determine whether SPINK4 can affect ferroptosis in CRC cells in vivo, HCT116 cells transfected with pcDNA3.1-SPINK4 were implanted into the subcutaneous space of nude mice, and the empty vector was used as a control. The results indicated that compared with the NC control group, overexpression of SPINK4 significantly increased tumor size. Moreover, although Erastin treatment decreased tumor size, the overexpression of SPINK4 also significantly promoted tumor size in the presence of Erastin (Fig. 5A-C), suggesting that the overexpression of SPINK4 blocked Erastin-induced ferroptosis in vivo.

## Discussion

SPINK4 is a member of the SPINK protease inhibitor family and acts as an important mediator of many vital processes in the mammalian body [22]. A previous study reported that SPINK4 was overexpressed in the intestinal epithelial cells in active celiac disease compared to that in inactive celiac disease [23]. Expression of SPINK4 was also found to be significantly increased in the colon mucosa of an inflammatory bowel disease rat model compared with that in normal rats [24]. Furthermore, in a three-layer epigenome-wide association study, SPINK4 was identified as a risk locus for ulcerative colitis [25], and a single-cell RNA sequence study revealed that SPINK4 was associated with Barrett's esophagus [26]. Collectively, current studies have demonstrated that aberrant expression of SPINK4 is involved in gastrointestinal diseases.

Although SPINK4 has been implicated in several diseases, little is known regarding its role in CRC. Currently, only a few studies have explored the expression of SPINK4 in CRC and found that SPINK4 downregulation is associated with poor survival in CRC patients [15]; however, inconsistent results have been reported that increased SPINK4 expression was related to poor outcomes in rectal cancer patients [16]. These inconsistencies suggest that the association between SPINK4 and CRC is yet to be validated. In the present study, by analyzing data from eight GEO datasets, which included larger samples, we observed that SPINK4 was significantly decreased in CRC tissues compared with adjacent normal tissues. In addition, the IHC assay results indicated similar results regarding the SPINK4 protein, which in in agreement with a previous report [15]. Considering the fewer samples in previous studies [15, 16], the present study may further reveal that SPINK4 expression is reduced as CRC progresses and possibly acts as a crucial molecule in the pathogenesis of CRC.

Since the biological function of SPINK4 in CRC cells is still unknown, we investigated it by altering its expression in these cells. We overexpressed SPINK4 in LoVo and HCT116 cells since its expression was lower in CRC tissues relative to normal tissues. However, four CRC cell lines express SPINK4 at levels higher than the human colon mucosal epithelial cell line NCM460, and overexpression of SPINK4 greatly aided LoVo and HCT116 cells in proliferating and metastasizing. Furthermore, the animal model demonstrated that SPINK4 overexpression enlarged tumors. Overall, these in vivo and in vitro tests showed that SPINK4 promotes the growth and metastasis of CRC cells. These findings suggest that SPINK4 plays a tissue-specific role.

Results of our study suggest that SPINK4 played a conflicting role in tissues and CRC cells. We hypothesized that the opposite results could be attributed to the following factors: first, there was much greater heterogeneity in CRC tissues than in CRC cells, and the results in CRC tissues reflected SPINK4 expression across the board, whereas the results in CRC cells only reflected a small portion of CRC; and second, SPINK4 expression may have been controlled by other factors in tissues but only had a minor impact on cell lines. Third, even though there are still many CRC cell lines, we only examined the expression of SPINK4 in four of them. Nonetheless, this expression may be consistent with that seen in tissues. Third, we only tested the expression of SPINK4 in four CRC cells, there are many CRC cells lines, which the expression might in agreement with that in tissues. Thus, it is important to examine the precise expression of SPINK4 in CRC at single-cell levels or in larger numbers of CRC cells.

Recently, growing evidence has demonstrated the potential of triggering ferroptosis in cancer therapy, particularly for the eradication of aggressive tumors that are resistant to traditional therapies [27, 28]. Although there is evidence of association of several cytokines with ferroptosis in some diseases, there is no prior study looking at the role of SPINK4 in ferroptosis in CRC. In the present study, we found that SPINK4 was significantly reduced after ferroptosis was induced in CRC cells, indicating that SPINK4 could inhibit ferroptosis in CRC. The cell immunofluorescence assay revealed that SPINK4 located in both the nucleoplasm and nucleus of CRC cells, where the expression of SPINK4 in the nucleus was more prominent after treatment with Erastin, suggesting that SPINK4 plays a role in the nucleus after ferroptosis. By overexpressing SPINK4 in CRC cells and evaluating the change in ferroptosis, we further revealed that SPINK4 inhibits ferroptosis in CRC cells. Finally, nude mouse model experiments confirmed that SPINK4 promotes tumor growth and suppresses the ferroptotic effect, demonstrating that SPINK4 may act as an inhibitor of ferroptosis in CRC cells.

Despite the present study determined the role of SPINK4 in CRC cells and its effect on ferroptosis, our study did not elucidate the mechanism underlying SPINK4-regulated CRC cell functioning and the pathway related to ferroptosis needs to be determined.

Little is known about the role of SPINK4 in controlling disease yet. Only one investigation found that increasing SPINK4 was associated with reduced IgG galactosylation [29]. We hypothesized that SPINK4 exerts its biological function by interacting with molecules that affect ferroptosis, such as GSH, GPX4, or other molecules that regulate ferroptosis [30], because SPINK4 protein was primarily found in the nucleoplasm and nucleus of CRC cells. Future research should therefore focus on the molecules that SPINK4 interacts with downstream in CRC cells in order to better understand SPINK4 function in CRC.

## Conclusions

In this study, we revealed that SPINK4 expressed in CRC tissues was decreased and that the proliferation and metastasis of CRC were driven by the enhanced expression of SPINK4. We also found that SPINK4 was able to inhibit CRC ferroptosis; however, the underlying mechanism needs to be explored in future studies.

## Supplementary Information

**Additional file 1.**

## Data Availability

The data used to support the findings of this study are available from GEO databases(www.ncbi.nlm.nih.gov/geo/), with the access number as GSE39582, GSE106582, GSE83889, GSE50421, GSE21510, GSE81558, GSE21815, and GSE32323, respectively. The TCGA-COADREAD datasets was downloaded from Xena database (https://xenabrowser.net/datapages/).
