# Supplementary material for: SPINK4 promotes colorectal cancer cell proliferation and inhibits ferroptosis
Source: BMC Gastroenterol. 2023 Apr 3;23:104. doi: 10.1186/s12876-023-02734-2 (PMC10071753; doi:10.1186/s12876-023-02734-2)
Supplement: Supplementary file 1 — Additional file 1. [file 12876_2023_2734_MOESM1_ESM.pdf]

Original western-blot graph

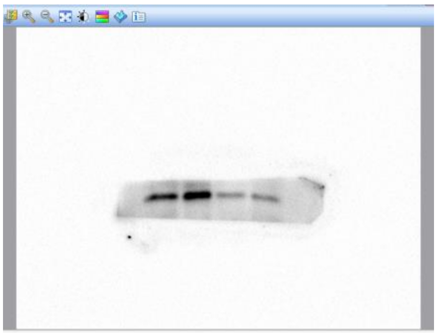

Figure 2B SPINK4

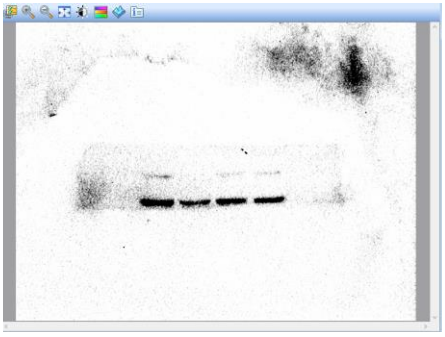

Figure 2B β-actin

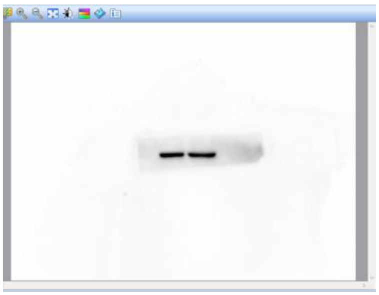

Figure 3B β-actin

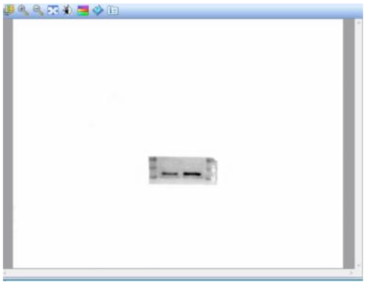

Figure 3B GPX4-HCT116

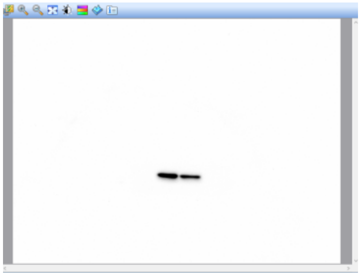

Figure 3B GPX4-LOVO

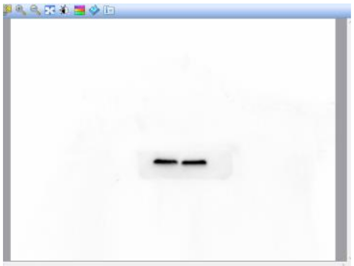

Figure 3E β-actin

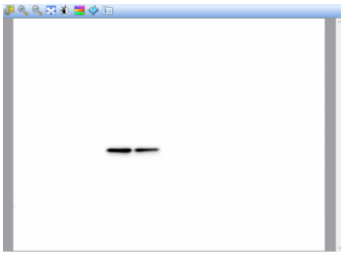

Figure 3E SPINK4-HCT116

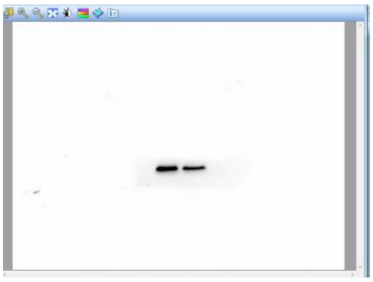

Figure 3E SPINK4-LOVO

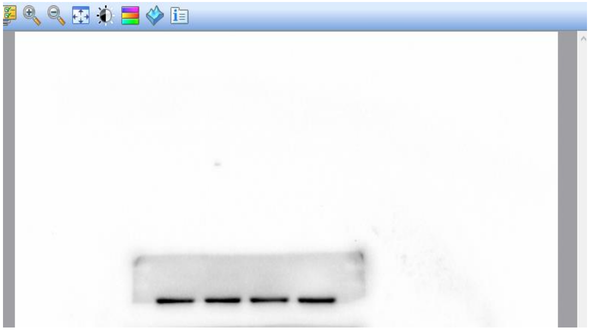

Figure 4B β-actin

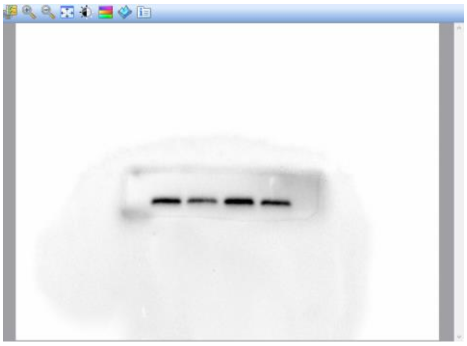

Figure 4B GPX4

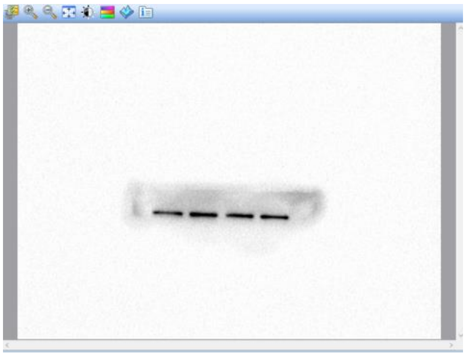

Figure 4D β-actin

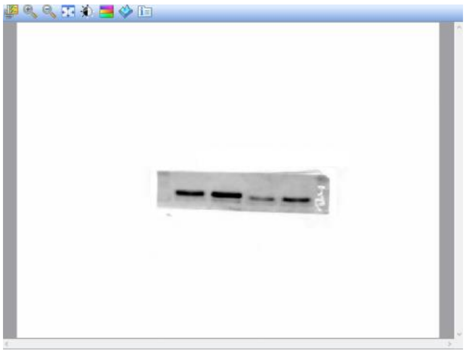

Figure 4D GPX4
